# The Effects of Florfenicol on the Values of Serum Tumor Necrosis Factor-**α** and Other Biochemical Markers in Lipopolysaccharide-Induced Endotoxemia in Brown Trout

**DOI:** 10.1155/2014/464373

**Published:** 2014-04-29

**Authors:** Ayse Er, Burak Dik

**Affiliations:** Department of Pharmacology and Toxicology, Faculty of Veterinary Medicine, University of Selçuk, 42075 Konya, Turkey

## Abstract

The aim of the present study was to determine the effects of florfenicol on the expected changes in sTNF-**α**, damage markers of the liver and kidney, and the lipid metabolism parameters in endotoxemic brown trout. Ninety-six brown trout were included in this study. After six of the fish were reserved as the control group, the remaining 90 fish were divided equally into 3 groups as follows: LPS (2 mg/kg, IP), LPS (2 mg/kg, IP) + florfenicol (40 mg/kg, IM), and florfenicol (40 mg/kg, IM). Blood samples were obtained from the tail of the fish at 1.5, 3, 6, 10, and 24 hours. The levels of sTNF-**α** were determined by ELISA and biochemical markers were evaluated with an autoanalyzer. A significant increase was observed in the values of sTNF-**α** in the LPS and LPS + florfenicol groups (*P* < 0.05). Significant increases were found in the kidney and liver damage determinants in the LPS and LPS + florfenicol groups (*P* < 0.05). Irregular changes in the lipid metabolism parameters were observed in all the subgroups. In conclusion, florfenicol does not affect the increases of sTNF-**α** caused by LPS and does not prevent liver or kidney damage; at least, it can be said that florfenicol does not have any evident positive effects on the acute endotoxemia of fish.

## 1. Introduction

Florfenicol (d-threo chloramphenicol) is a broad spectrum antibiotic that belongs to the amphenicol group that inhibits protein synthesis through its bacteriostatic activity. Its spectrum of effect includes many gram negative and positive bacteria including* Escherichia coli (E. coli)*,* Salmonella* species (*Salmonella* sp.),* Pasteurella* sp.,* Shigella* sp.,* Bordetella* sp.,* Chlamydia* sp., and* Mycoplasma* species. Although florfenicol is an analog of chloramphenicol, it has two main structural differences. It includes a p-methyl sulfonyl group in place of the p-nitro group seen in chloramphenicol and a fluorine atom instead of a hydroxyl group. The use of florfenicol in cows, pigs, chickens, and fish has been approved by the European Medicines Agency (EMEA) [1, 2]. It is recommended in the treatment of bacterial infections caused by* Vibrio anguillarum, Edwardsiella *sp., and* Flavobacterium* sp. in fish [3]. It has been reported that florfenicol can be safely used at differing doses in tilapia fish and dose related mild decreases in the hematopoietic/lymphopoietic tissues have been observed with its use in channel catfish [4].

Lipopolysaccharide (LPS, endotoxin), which is a component in the wall of gram negative bacteria, is quite dangerous for living organisms [5]. LPS may be used in experimental modelling to either induce a model of local inflammation [6] or generate a model of systemic endotoxemia [7]. Induction of cytokine synthesis and alterations in complete blood count (haemogram), markers of organ damage, and biochemical parameters related to lipid metabolism may be observed in organisms in response to the application of LPS [7–9]. LPS-induced endotoxemia may lead to a variety of clinical manifestations including fever, multiple organ failure, septic shock, and death [5, 10]. In living organisms, LPS is recognized by immune cells and, as a result, the immune system is activated [5]. Activated cells synthesize a number of cytokines including sTNF-*α* and interleukins [8, 11]. TNF-*α* alone has the ability to initiate the pathology of sepsis such as decreased blood pressure (hypotension), multiple organ insufficiency, and fever [10]. It has been determined in mice that florfenicol decreases the levels of interleukin-4, -5, and -13 (IL-4, -5, and -13) and can have an anti-inflammatory effect [12].

Drugs can also have a number of clinical and biochemical side-effects that may present as alterations in the values of some biochemical parameters from both the plasma and serum. These biochemical changes are accepted as warnings for the start of structural damage in some tissue and organs [13, 14]. While increases in alkaline phosphatase (ALP) are expected in damage of the biliary tract, values of alanine aminotransferase (ALT) and aspartate aminotransferase (AST) are determined to be elevated in liver damage. With insufficiencies of the livers synthesis ability, changes in the levels of serum total protein and albumin can be determined. Increased levels of blood urea nitrogen (BUN) and creatinine in the serum demonstrate a decline in kidney function. Serum high density lipoprotein (HDL), low density lipoprotein (LDL), and triglyceride (TG) values provide information regarding lipid metabolism [15–17].

In this study, it was hypothesized that the application of LPS in fish and the resulting endotoxemia would increase the levels of both sTNF-*α* and organ damage markers as is observed in mammals [7, 9] and would cause changes in lipid metabolism [8] and these changes would be improved with florfenicol, which has effects on the immune system [12].

The aim of the present study was to determine the effects of florfenicol on the expected changes in serum of sTNF-*α*, liver (ALP, AST, ALT, total protein, and albumin) and kidney (BUN and creatinine) damage markers, and lipid metabolism parameters (TG, HDL, and LDL) in the endotoxemia induced with LPS in brown trout.

## 2. Materials and Methods

### 2.1. Animals

Ninety-six brown trout (166–267 g, Dumlupinar fish facility, Bozkir, Konya, Turkey) were used in this study. The presented study was organized in accordance with the guidelines provided by Selçuk University, Experimental Medicine, Research and Application Center, Konya, Turkey. The fish were kept in tanks with fresh open water circuits under natural conditions of light and temperature. The fish were fed daily with commercial trout pellets (Blueaq, Abalioglu, Turkey).

### 2.2. Experimental Procedure

Following the randomized separation of six fish for the control group, the rest of the fish were divided into three equal groups: the LPS (*E. coli*, serotype O111: B4, Sigma-Aldrich Chemie, Deisenhofen, Germany, 2 mg/kg, IP) group, the LPS (2 mg/kg IP) + florfenicol (Nuflor flk, Intervet Ilac, Istanbul, 40 mg/kg, IM) group, and the florfenicol (40 mg/kg, IM) group, respectively. Following these applications, the fish were anesthetized with quinaldine (25 mg/L) for anesthesia and blood samples were taken from their tails at 1.5, 3, 6, 10, and at 24 hours.

### 2.3. Measurements

The serum samples were kept at −70°C until analysis. The sTNF-*α* levels were measured by commercial test kit, and analytical procedure were performed according to the manufacturer recommendations. The ELISA plate reader (MWGt Lambda Scan 200, USA) was used to evaluate the value of sTNF-*α* and an autoanalyzer (ILab-300 bioMérieux Diagnostics, Milan, Italy) was used to measure biochemical parameters including ALP, AST, ALT, total protein, albumin, BUN, creatinine TG, HDL, and LDL.

### 2.4. Statistical Analysis

Findings were statistically evaluated by the one-way ANOVA followed by Duncan post hoc test (SPSS 19.0). *P* values lower than 0.05 (*P* < 0.05) were accepted as being statistically significant.

## 3. Results

The effects of florfenicol on the sTNF-*α* and biochemical parameters of the endotoxemic fish are presented in Table 1. The sTNF-*α* values were found to be significantly increased in the LPS (1.5 and 3 hours) and LPS + florfenicol (1.5 hour) groups compared to the control hour (0 hour) (*P* < 0.05).

While ALP, AST, and ALT values were found to be increased in the LPS group (*P* < 0.05), ALT and AST values were increased in the LPS + florfenicol group only (*P* < 0.05). It was determined that florfenicol significantly increased the values of ALP, AST, and ALT in healthy fish (*P* < 0.05). There were a significant elevation in total protein values (*P* < 0.05) and a reduction in albumin levels (*P* < 0.05) in the LPS group and a significant decrease in the values of total protein in the florfenicol group (*P* < 0.05). LPS application was determined to significant increase serum BUN and creatinine (*P* < 0.05), whereas, with LPS + florfenicol application, increases were observed only in creatinine values (*P* < 0.05).

In the LPS group, HDL, LDL, and TG levels were increased (*P* < 0.05). In the LPS + florfenicol group, HDL levels were found to be increased (*P* < 0.05) and, in the florfenicol group, HDL and LDL levels were increased (*P* < 0.05) and TG levels were decreased (*P* < 0.05).

## 4. Comment

Florfenicol is a broad spectrum antibiotic used for the treatment of bacterial infections in mammals, birds, fish, and lobsters [1, 2, 4, 18, 19]. In this study, sTNF-*α* values were found to increase after LPS application (*P* < 0.05) (Table 1). LPS induces production of inflammation mediators such as cytokines by stimulating the transcription factor nuclear factor-kappaB (NF-*κ*B) in cells [8, 20] and, after LPS application to fish, TNF-*α* levels have been reported to increase [8]. We determined that LPS-induced production of sTNF-*α* could not be suppressed by the administration of florfenicol in brown trout (Table 1).

Some antibiotics have a suppressive effect on the increases of sTNF-*α* [21, 22]. Florfenicol suppresses humoral and cellular immune responses in mice [23, 24] and reduces the levels of sTNF-*α* by inhibiting the efficiency of NF-*κ*B [25, 26]. Moreover, florfenicol has an anti-inflammatory and a dose-dependent downregulatory effect on the synthesis of some interleukins [12]. Florfenicol also inhibits the synthesis of prostaglandin E2 [27]. In the present study, the reasons for not determining sTNF-*α* suppression with florfenicol may be due to the used dose of florfenicol or differences in the animal species. We determined that florfenicol applied alone does not have any effect on the sTNF-*α* levels in healthy brown trouts (Table 1). Although it is known that antibiotics affect the synthesis of cytokines in living organisms [21, 28], Lunden et al. reported that florfenicol does not have a significant influence on antibody synthesis and leukocyte number in rainbow trout [29].

In this study, it was determined that LPS application significantly increases levels of the serum liver damage markers (ALP, ALT, and AST) (*P* < 0.05) and, with the application of LPS + florfenicol, there was no decrease in the elevated liver damage markers (ALT and AST) except for ALP (Table 1). In addition, LPS was found to affect protein synthesis, which is accepted as being a parameter of the synthesis ability of the liver (Table 1).

Swain et al. [8] determined an accumulation of LPS both in the liver and in the kidney shortly after its intravenous (IV) application in fish. Additionally, an increase in the expression of damage markers of the liver was determined in response to the administration of LPS [6, 9]. These results suggest that LPS could cause hepatotoxicity in fish as it does in mammals.

It was noticed that the administration of florfenicol in healthy fish did cause increases in the liver damage markers (AST and ALT) (*P* < 0.05) (Table 1). It has been reported that many antibiotics [30] as well as amphenicols [31] are hepatotoxic and florfenicol can cause an increase in the weight of the liver [32]. A twofold increase in the values of the serum ALP is accepted as cholestatic damage while a twofold increase in serum ALT is synonymous for hepatocellular damage. When it is taken into consideration that, in drug related liver damage, treatment is terminated when ALT levels are increased 5 times those of normal [33], it can be said that florfenicol does not have a high potential for liver damage in brown trout.

In this study, while the markers of kidney damage (BUN and creatinine) were increased in the LPS group (*P* < 0.05), only an increase in the level of creatinine (*P* < 0.05) was observed in the LPS + florfenicol group. It has been reported that LPS accumulates quickly in the kidney of fish after intravenous administration [8] and kidney damage markers are increased in endotoxemia [9, 21]. The basic reason for kidney damage in LPS-induced endotoxemia has been shown to be due to hemodynamic changes resulting in the reduction in glomerular filtration rate, microcoagulation, and hypoxic conditions [34]. We observed a temporary decrease in creatinine levels after the administration of florfenicol in healthy fish (*P* < 0.05) (Table 1). It is a known fact that antibiotics like a number of other drugs may have effects on kidney function [35].

We determined that the administration of LPS results in an increase in the levels of HDL, LDL, and TG (*P* < 0.05). It was determined that, in endotoxemic fish, the administration of florfenicol corrected the changes in LDL and TG values but had no effect on the elevated HDL levels (Table 1). Swain et al. [8] reported that LPS can affect the parameters of lipid metabolism, but these effects can differ depending on the type of LPS and the dose given. It was observed that the value of TG was usually increased whereas the values of LDL and HDL were decreased. In rodents, it has been stated that lipoproteins can demonstrate a protective effect against the deaths due to LPS [36]. Fish are more resistant to endotoxic shock than humans and other animals [8]. This may be due to the different physiological responses that living organisms may express to LPS.

It was determined that florfenicol led to increases in the levels of both HDL and LDL (*P* < 0.05) and decreases in the values of TG (*P* < 0.05) in healthy fish (Table 1). There was no source material found on the effects of florfenicol on lipid metabolism in animals. However, it has been reported that antibiotics affect lipid metabolism [37] and florfenicol causes changes in hematological parameters [32].

In conclusion, it can be stated that, in endotoxemic brown trout, sTNF-*α* synthesis is stimulated, damage develops in the liver and kidney, lipid metabolism is affected, with florfenicol, these changes do not disappear, and when florfenicol is given to healthy fish, it can affect the functions of the kidney and liver and the metabolism of lipids.

## Figures and Tables

**Table 1:** The effect of florfenicol (F) on sTNF-*α* and serum biochemical indices in endotoxemic fish (mean ± SE).

	Groups	0 hour	1.5 hour	3 hours	6 hours	10 hours	24 hours
TNF-*α* (pg/mL)	LPS	728 ± 188^b^	2064 ± 244^a^	2094 ± 474^a^	1022 ± 412^ab^	1642 ± 606^ab^	736 ± 232^b^
	LPS + F	728 ± 188^b^	2313 ± 321^a^	810 ± 425^b^	1254 ± 440^ab^	1101 ± 480^ab^	1876 ± 536^ab^
	F	728 ± 188^a^	1681 ± 545^a^	1462 ± 439^a^	1196 ± 264^a^	976 ± 431^a^	1208 ± 228^a^

ALP (U/L)	LPS	90.0 ± 11.4^b^	87.0 ± 16.8^b^	87.0 ± 11.1^b^	160 ± 15.0^a^	94.0 ± 7.77^b^	123 ± 11.7^b^
	LPS + F	90.0 ± 11.4^a^	93.0 ± 13.0^a^	90.0 ± 5.46^a^	128 ± 18.7^a^	116 ± 17.3^a^	103 ± 13.8^a^
	F	90.0 ± 11.4^ab^	82.0 ± 17.0^b^	109 ± 23.2^ab^	132 ± 12.9^a^	107 ± 13.5^ab^	106 ± 3.57^ab^

ALT (U/L)	LPS	4.50 ± 0.43^b^	4.83 ± 0.95^b^	4.67 ± 0.49^b^	18.7 ± 4.70^a^	8.83 ± 2.18^b^	4.83 ± 0.31^b^
	LPS + F	4.50 ± 0.43^d^	7.17 ± 1.11^cd^	10.3 ± 0.72^bcd^	14.8 ± 2.24^ab^	18.2 ± 4.37^a^	12.7 ± 2.01^abc^
	F	4.50 ± 0.43^c^	9.67 ± 1.15^ab^	7.67 ± 0.92^bc^	12.8 ± 2.20^a^	12.2 ± 0.95^a^	11.5 ± 1.09^a^

AST (U/L)	LPS	223 ± 27.3^c^	220 ± 27.2^c^	242 ± 16.0^bc^	477 ± 51.6^a^	323 ± 25.2^b^	325 ± 18.0^b^
	LPS + F	223 ± 27.3^c^	281 ± 41.5^c^	343 ± 33.9^bc^	469 ± 43.7^a^	405 ± 54.8^ab^	466 ± 30.7^a^
	F	223 ± 27.3^d^	344 ± 44.5^bc^	285 ± 12.3^cd^	499 ± 48.5^a^	440 ± 34.5^ab^	523 ± 41.3^a^

Total protein (g/dL)	LPS	3.80 ± 0.48^b^	5.40 ± 0.51^a^	3.70 ± 0.31^b^	3.60 ± 0.30^b^	3.40 ± 0.36^b^	3.30 ± 0.08^b^
	LPS + F	3.80 ± 0.48^a^	3.40 ± 0.38^a^	3.40 ± 0.14^a^	3.10 ± 0.17^a^	3.70 ± 0.26^a^	3.30 ± 0.15^a^
	F	3.80 ± 0.48^a^	3.00 ± 0.21^ab^	3.20 ± 0.13^ab^	2.70 ± 0.18^b^	3.30 ± 0.25^ab^	3.30 ± 0.13^ab^

Albumin (g/dL)	LPS	1.90 ± 0.12^a^	1.90 ± 0.08^a^	1.50 ± 0.03^b^	1.90 ± 0.14^a^	1.70 ± 0.10^ab^	1.80 ± 0.05^a^
	LPS + F	1.90 ± 0.12^a^	2.00 ± 0.24^a^	1.70 ± 0.03^a^	1.80 ± 0.02^a^	2.00 ± 0.09^a^	1.80 ± 0.05^a^
	F	1.90 ± 0.12^a^	1.70 ± 0.14^a^	1.70 ± 0.07^a^	1.90 ± 0.22^a^	1.90 ± 0.07^a^	1.80 ± 0.09^a^

Creatinine (mg/dL)	LPS	0.26 ± 0.03^cd^	0.93 ± 0.12^a^	0.38 ± 0.02^bc^	0.51 ± 0.03^b^	0.35 ± 0.03^c^	0.15 ± 0.01^d^
	LPS + F	0.26 ± 0.03^bc^	0.22 ± 0.01^cd^	0.20 ± 0.02^cd^	0.38 ± 0.03^a^	0.33 ± 0.04^ab^	0.17 ± 0.01^d^
	F	0.26 ± 0.03^a^	0.21 ± 0.02^ab^	0.20 ± 0.01^ab^	0.16 ± 0.01^b^	0.21 ± 0.02^ab^	0.22 ± 0.02^ab^

BUN (mg/dL)	LPS	4.50 ± 0.67^b^	6.67 ± 0.99^a^	3.83 ± 0.40^b^	6.83 ± 0.60^a^	4.33 ± 0.80^b^	4.33 ± 0.33^b^
	LPS + F	4.50 ± 0.67^a^	5.83 ± 1.72^a^	3.83 ± 0.48^a^	6.33 ± 0.76^a^	3.50 ± 0.56^a^	3.83 ± 0.95^a^
	F	4.50 ± 0.67^a^	5.17 ± 0.70^a^	4.67 ± 0.42^a^	5.83 ± 0.91^a^	6.00 ± 0.68^a^	6.00 ± 0.58^a^

HDL (mg/dL)	LPS	123 ± 3.61^bc^	101 ± 7.34^c^	108 ± 8.00^c^	151 ± 9.33^a^	132 ± 9.34^ab^	147 ± 5.69^a^
	LPS + F	123 ± 3.61^bc^	109 ± 10.7^c^	140 ± 5.49^ab^	133 ± 5.43^ab^	152 ± 6.41^a^	131 ± 7.70^ab^
	F	123 ± 3.61^b^	131 ± 11.1^b^	134 ± 3.46^b^	193 ± 18.8^a^	151 ± 2.31^b^	150 ± 5.71^b^

LDL (mg/dL)	LPS	35.0 ± 1.67^bc^	39.0 ± 4.95^bc^	32.0 ± 2.47^c^	46.0 ± 4.62^ab^	39.0 ± 2.18^bc^	51.0 ± 4.30^a^
	LPS + F	35.0 ± 1.67^a^	34.0 ± 3.20^a^	46.0 ± 5.16^a^	48.0 ± 6.21^a^	47.0 ± 3.94^a^	39.0 ± 3.75^a^
	F	35.0 ± 1.67^bc^	33.0 ± 5.00^c^	35.0 ± 3.77^bc^	46.0 ± 1.38^a^	42.0 ± 3.23^abc^	44.0 ± 2.35^ab^

TG (mg/dL)	LPS	377 ± 55.3^b^	562 ± 95.1^a^	243 ± 19.7^b^	340 ± 59.9^b^	245 ± 23.1^b^	239 ± 23.1^b^
	LPS + F	377 ± 55.3^a^	369 ± 66.1^a^	241 ± 33.6^a^	249 ± 20.5^a^	291 ± 33.9^a^	257 ± 21.7^a^
	F	377 ± 55.3^a^	194 ± 25.6^c^	284 ± 34.2^abc^	340 ± 42.1^ab^	185 ± 16.1^c^	260 ± 26.2^bc^

LPS: lipopolysaccharide, F: florfenicol, TNF-*α*: tumor necrosis factor alpha, ALP: alkaline phosphatase, ALT: alanine aminotransferase, AST: aspartate aminotransferase, BUN: blood urea nitrogen, HDL: high density lipoproteins, LDL: low density lipoproteins, TG: triglycerides, and ^a, b, c, d^different letters on the same line refer to statistical significance (*P* < 0.05).
